# Association of serum calcium and heart failure with preserved ejection fraction in patients with type 2 diabetes

**DOI:** 10.1186/s12933-016-0458-6

**Published:** 2016-10-03

**Authors:** Junfeng Li, Nan Wu, Wenling Dai, Liu Jiang, Yintao Li, Shibao Li, Zhongyuan Wen

**Affiliations:** 1 Department of Endocrinology, Renmin Hospital of Wuhan University, Wuhan, 430060 China; 2 Department of Geriatrics, Zhongshan Hospital of Fudan University, Shanghai, 200032 China; 3 Department of Medical Oncology, Shandong Cancer Hospital and Institute, Shandong University School of Medicine, Jinan, 250012 China; 4 Department of Medical Laboratory, The Affiliated Hospital of Xuzhou Medical College, No. 99 Huaihai West Road, Xuzhou, 221000 China

**Keywords:** Calcium, Heart failure with preserved ejection fraction, Type 2 diabetes mellitus

## Abstract

**Background:**

Type 2 diabetes mellitus (T2DM) is a recognized trigger factor for heart failure with preserved ejection fraction (HFpEF). Recent studies show that higher serum calcium level is associated with greater risk of both T2DM and heart failure. We speculate that increased serum calcium is related to HFpEF prevalence in patients with T2DM.

**Methods:**

In this cross-sectional echocardiographic study, 807 normocalcemia and normophosphatemia patients with T2DM participated, of whom 106 had HFpEF. Multinomial logistic regression was carried out to determine the variables associated with HFpEF. The associations between serum calcium and metabolic parameters, as well as the rate of HFpEF were examined using bivariate linear correlation and binary logistic regression, respectively. The predictive performance of serum calcium for HFpEF was evaluated using the area under the receiver operating characteristic curve (AUC).

**Results:**

Patients with HFpEF have significantly higher serum calcium than those without HFpEF. Serum calcium was positively associated with total cholesterol, triglycerides, low-density lipoprotein cholesterol, serum uric acid, HOMA-IR and fasting plasma glucose. Compared with patients in the lowest serum calcium quartile, the odds ratio (OR) for HFpEF in patients in the highest quartile was 2.331 (95 % CI 1.088–4.994, p = 0.029). When calcium was analyzed as a continuous variable, per 1 mg/dL increase, the OR (95 % CI) for HFpEF was [2.712 (1.471–5.002), p = 0.001]. Serum calcium can predict HFpEF [AUC = 0.673, 95 % CI (0.620–0.726), p < 0.001].

**Conclusions:**

An increase in serum calcium level is associated with an increased risk of HFpEF in patients with T2DM.

## Background

Heart failure (HF) is magnified in individuals with type 2 diabetes mellitus (T2DM), in whom incidence rates are 2–5 times greater than those in the general population [1, 2]. Heart failure with preserved ejection fraction (HFpEF) constitutes approximately 50–55 % of the HF population [3] and the prevalence of HFpEF is rising at a rate of around 1 % per year [4], so it is predicted that HFpEF will become the most prevalent phenotype of HF over the next decade [4, 5]. Despite robust evidence of prognostic benefit using therapies with angiotensin-converting enzyme inhibitors, angiotensin-1 receptor blockers and β-blockers in heart failure with reduced ejection fraction (HFrEF), all outcome trials in HFpEF to date have failed to demonstrate survival benefit [3, 6]. Since much less is known about the pathophysiology and treatment of HFpEF in contrast to HFrEF [5, 6], screening potential risk factors in the progression of HFpEF in diabetic patients is of particular importance.

Recent studies from clinical electrophysiology and preclinical experiments have demonstrated that abnormal intracellular calcium homeostasis is a key determinant in HFpEF [5, 7, 8]. Meanwhile, cumulative evidences reveal that an increase in serum calcium level is independently associated with increased risk of T2DM [9, 10] and cardiovascular disease [11] even in normocalcemic populations.

Based on these findings, we speculate that an alteration in serum calcium is associated with HFpEF prevalence, and we conduct a cross-sectional study to evaluate relationships between serum calcium levels and HFpEF in T2DM patients.

## Methods

### Participants

A total of 807 subjects (463 men and 344 women) were included in this study. We recruited consecutive subjects aged 40 years or older who visited Renmin Hospital for education, evaluation, or treatment of T2DM from 2012 to 2015.

To minimize the possibility that some abnormal conditions may influence the results, patients with any of the following conditions were excluded: (1) history of left ventricular ejection fraction (LVEF) <50 % at any time; (2) isolated right heart failure due to pulmonary disease; (3) dyspnoea due to non-cardiac causes such as pulmonary disease, anaemia, or severe obesity; (4) primary valvular or myocardial diseases, atrial fibrillation, coronary artery or cerebrovascular disease needing revascularisation within 3 months; (5) serum creatinine >130 μmol/L (normal range: 50–130 μmol/L) or urine albumin per gram urine creatinine (Alb/Cr) >300 mg/g; (6) uncontrolled thyroid diseases, history of parathyroid disease or vitamin D-related disorders; (7) medication history including vitamin D, bisphosphonate, estrogen replacement therapy and diuretics which may influence calcium metabolism within the past 1 month; (8) serum calcium out of normal range from central laboratory of Renmin hospital (8.42–10.42 mg/dL, or 2.10–2.60 mmol/L); (9) serum phosphate out of normal range from central laboratory of Renmin hospital (3.00–4.50 mg/dL, or 0.97–1.45 mmol/L).

HFpEF was diagnosed according to the European Society of Cardiology guideline [12]: (1) presence of symptoms and/or signs of HF; (2) LVEF ≥50 %; (3) NT-proBNP >125 pg/mL. T2DM was diagnosed by the American Diabetes Association guideline [13]. Obesity was defined as body mass index (BMI) ≥28 kg/m^2^ according to Chinese standard [14]. Dyslipidemia was defined as HDL-C <1.04 mmol/L, LDL-C ≥4.14 mmol/L, or TG ≥2.26 mmol/L [15]. Smoking was defined as “ever smoked” as compared to “never smoked”. Hypertension was defined as systolic blood pressure (SBP) ≥140 mmHg and/or diastolic blood pressure (DBP) ≥90 mmHg, or current antihypertensive therapy. Micro-albuminuria was defined as Alb/Cr between 30 and 300 mg/g, and macro-albuminuria was defined as Alb/Cr >300 mg/g.

This study was approved by the ethical review board of Renmin Hospital and complied with the Helsinki declaration. Written informed consent was obtained from all participants.

### Biochemical measurements

A 12-h overnight fasting venous blood sample was collected in all subjects. A first morning urine sample was collected once a day for 3 consecutive days to estimate the Alb/Cr. The calcium, phosphate, uric acid, creatinine, albumin, total cholesterol (TC), triglycerides (TG), low-density lipoprotein cholesterol (LDL-C), high-density lipoprotein cholesterol (HDL-C), and fasting plasma glucose (FPG) were measured by biochemical auto analyzer (Abbott C8000). Measurements of insulin and NT-proBNP were performed by immunoassay technique on the Roche Elecsys 2010 systerm. HbA1c was measured by high performance liquid chromatography (HPLC; Bio-Rad, Hercules, CA, USA). Serum calcium level was corrected according to the formula: albumin-adjusted serum calcium concentration (mg/dL) = measured serum calcium concentration (mg/dL) + 0.8 × [4 − serum albumin concentration (g/dL)] [10]. Insulin resistance was assessed by the homeostatic model: HOMA-IR = fasting plasma glucose (mmol/L) × fasting plasma insulin (mIU/L)/22.5 [16].

### Echocardiography

According to the American Society of Echocardiography [17], with patients in partial left lateral decubitus positions, echocardiographic examinations were performed under two-dimensional guided M-mode with a Vingmed System 5 Doppler echocardiographic unit (GE Vingmed Ultrasound, Horten, Norway). Left ventricular mass (LVM) was calculated by the Devereux formula [18]: LVM (g) = 0.8{1.04 [([LVIDD (left ventricular internal diameter, diastolic) + PWTD (posterior wall thickness, diastolic) + IVSD (inter ventricular septum, diastolic)]^3^ − LVIDD^3^)]} + 0.6. Relative wall thickness (RWT) was calculated as 2 × PWTD/LVIDD and increased RWT was defined as >0.42 [19]. LVM index (LVMI) was derived by correcting LVM for body surface area [BSA (m^2^) = 0.007184 × height(cm)^0.725^ × weight(kg)^0.425^] [20]. LVH was defined as follows: LVMI >115 g/m^2^ for men and LVMI >95 g/m^2^ for women [19]. LV geometry was defined as “normal” (both RWT and LVMI normal), “concentric remodeling” (increased RWT but normal LVMI), “eccentric hypertrophy” (increased LVMI but normal RWT), and “concentric hypertrophy” (both LVMI and RWT increased) [19]. Left atrial diameter (LAD) and aortic root dimension (AOD) were also measured. LV systolic function was assessed by LVEF, and diastolic function was assessed by early to late mitral inflow velocity ratio (E/A) as well as deceleration time (DT).

### Data analysis

Continuous variables were presented as mean ± standard deviation (SD), as well as frequencies and percentages for categorical variables. Normal distribution was checked by Kolmogorov–Smirnov Test. HOMA-IR and Alb/Cr were logarithmically transformed to approximate normal distribution for analysis. Differences in normally distributed variables were determined by independent-samples T test or One-way ANOVA. If data were non-normally distributed or not met the homogeneity of variances, a nonparametric test was performed. Chi square tests were applied for categorical variables. Bivariate linear correlation (Pearson correlation) analysis was carried out to evaluate the associations between albumin-adjusted serum calcium and metabolic parameters. Backward stepwise multinomial logistic regression analysis was carried out to determine the variables associated with HFpEF and to estimate confounding factors possibly disturbing the relationship between serum calcium and HFpEF. Binary logistic regression analysis was performed using HFpEF as the dependent variable to analyze the association between serum calcium and HFpEF after adjusting for potential confounders. Odds ratios (OR) with 95 % confidence intervals (CI) were calculated for the relative risk of increased serum calcium level with HFpEF. The ability to predict HFpEF of albumin-adjusted serum calcium was evaluated using the area under the curve (AUC) in the receiver operating characteristic (ROC) curve. All statistical analysis were performed using Statistical Product and Service Solutions (SPSS) version 19.0. All tests were two-sided, p < 0.05 was considered statistically significant.

## Results

### Clinical characteristics

In this study, 807 patients with T2DM were included, 42.6 % were female, with a mean age of 69.3 ± 12.1 years. The average duration of T2DM was 10.2 ± 8.3 years. HFpEF, hypertension, dyslipidemia, albuminuria, and obesity were present in 106 (13.1 %), 417 (51.7 %), 434 (53.8 %), 460 (57.0 %) and 116 (14.4 %) patients, respectively.

Significant differences in albumin-adjusted serum calcium (8.96 ± 0.36 vs. 9.22 ± 0.44 mg/dL, p < 0.001) were observed between non-HFpEF and HFpEF group (Table 1). The patients with HFpEF had higher levels of NT-proBNP, lg HOMA-IR, serum uric acid, serum creatinine and lg Alb/Cr, longer duration of diabetes, greater percentage of female gender, micro-albuminuria and LVH (concentric hypertrophy, especially), as well as lower levels of serum albumin than those without HFpEF.

**Table 1:** Baseline characteristics of subjects categorized by HFpEF

Characteristics	HFpEF		p value
	No	Yes	
Age (years)	68.3 ± 12.3	75.8 ± 8.6	*<0.001*
Female, n (%)	287 (40.9)	57 (53.8)	*0.013*
Hypertension, n (%)	362 (51.6)	55 (51.9)	0.962
Smoking, n (%)	163 (23.3)	26 (24.5)	0.773
Duration of diabetes (year)	9.9 ± 8.0	12.4 ± 9.7	*0.013*
SBP (mmHg)	136.8 ± 19.6	138.1 ± 19.3	0.512
DBP (mmHg)	79.5 ± 11.1	77.6 ± 10.2	0.103
BMI (kg/m^2^)	24.3 ± 3.9	24.3 ± 3.1	0.946
Obesity, n (%)	102 (14.6)	14 (13.2)	0.713
Laboratory			
FPG (mmol/L)	7.77 ± 3.04	7.95 ± 3.80	0.589
Lg HOMA-IR	0.56 ± 0.33	0.67 ± 0.32	*0.001*
HbA1c (%)	8.16 ± 1.96	7.86 ± 1.77	0.134
Albumin (g/L)	38.91 ± 4.98	36.88 ± 4.85	*<0.001*
Uric acid (μmol/L)	331.77 ± 98.71	371.18 ± 113.06	*0.001*
Creatinine (μmol/L)	80.51 ± 23.69	94.04 ± 27.40	*<0.001*
lg Alb/Cr (mg/g)	1.68 ± 0.70	1.89 ± 0.67	*0.004*
Micro-albuminuria, n (%)	389 (55.5)	71 (67.0)	*0.026*
NT-proBNP (pg/mL)	189.86 ± 70.09	645.00 ± 264.26	*<0.001*
TG (mmol/L)	1.81 ± 1.11	2.03 ± 1.66	0.184
TC (mmol/L)	4.81 ± 1.15	4.74 ± 1.20	0.518
HDL-C (mmol/L)	1.11 ± 0.34	1.13 ± 0.53	0.728
LDL-C (mmol/L)	2.73 ± 0.84	2.66 ± 0.86	0.476
Dyslipidemia, n (%)	370 (52.8)	64 (60.4)	0.144
Albumin-adjusted calcium (mg/dL)	8.96 ± 0.36	9.22 ± 0.44	*<0.001*
Phosphate (mg/dL)	3.55 ± 0.45	3.57 ± 0.48	0.698
Calcium–phosphate product (mg^2^/dL^2^)	31.79 ± 4.44	32.44 ± 4.79	0.167
Echocardiographic characteristics			
LAD (mm)	37.65 ± 4.65	41.67 ± 5.77	*<0.001*
AOD (mm)	32.49 ± 3.33	32.46 ± 3.04	0.927
LVIDD (mm)	47.21 ± 4.23	48.10 ± 4.90	*0.048*
LVIDS (mm)	29.95 ± 3.49	31.35 ± 4.04	*0.001*
IVSD (mm)	10.60 ± 1.52	11.57 ± 1.90	*<0.001*
PWTD (mm)	9.76 ± 1.27	10.46 ± 1.59	*<0.001*
RWT	0.42 ± 0.07	0.44 ± 0.09	*0.01*
LVMI (g/m^2^)	99.30 ± 21.10	118.35 ± 30.79	*<0.001*
LVEF (%)	65.95 ± 5.35	66.86 ± 7.23	0.217
E/A	1.10 ± 0.21	0.91 ± 0.20	*<0.001*
DT (ms)	208.51 ± 54.79	265.70 ± 50.65	*<0.001*
LV geometry			
Normal, n (%)	309 (44.1)	22 (20.8)	*<0.001*
Concentric remodeling, n (%)	163 (23.3)	14 (13.2)	
Eccentric hypertrophy, n (%)	110 (15.7)	22 (20.8)	
Concentric hypertrophy, n (%)	119 (17.0)	48 (45.3)	
LVH, n (%)	229 (32.7)	70 (66.0)	*<0.001*

Italic values represent* p* < 0.05

### Serum calcium and metabolism-related parameters

Bivariate linear correlation analysis showed that albumin-adjusted serum calcium level was significantly and positively correlated with metabolism-related parameters including FPG (r = 0.205, p < 0.001), lg HOMA-IR (r = 0.143, p < 0.001), uric acid (r = 0.175, p < 0.001), TG (r = 0.104, p = 0.003), TC (r = 0.125, p < 0.001) and LDL-C (r = 0.099, p = 0.005) (Table 2).

**Table 2:** Correlation coefficients between albumin-adjusted serum calcium and metabolic parameters

	Albumin-adjusted serum calcium	
	r	p value
Age (year)	0.057	0.103
Duration of diabetes (year)	0.017	0.633
SBP (mmHg)	0.02	0.574
DBP (mmHg)	−0.015	0.671
BMI (kg/m^2^)	−0.004	0.899
FPG (mmol/L)	0.205	*<0.001*
Lg HOMA-IR	0.143	*<0.001*
HbA1c (%)	0.036	0.314
Uric acid (μmol/L)	0.175	*<0.001*
TG (mmol/L)	0.104	*0.003*
TC (mmol/L)	0.125	*<0.001*
HDL-C (mmol/L)	−0.046	0.188
LDL-C (mmol/L)	0.099	*0.005*

Italic values represent* p* < 0.05

### LV geometry

Table 3 showed echocardiographic characteristics categorized by albumin-adjusted serum calcium quartiles. From albumin-adjusted serum calcium quartile 1 (8.42–8.70 mg/dL) to quartile 4 (9.23–10.42 mg/dL), there was a significantly overall upward tendency of LVH (from 22.7 to 52.5 %, p < 0.001).

**Table 3:** Echocardiographic characteristics of subjects categorized by albumin-adjusted serum calcium quartiles

Echocardiographic characteristics	Albumin-adjusted calcium concentration (mg/dL)				p value
	8.42–8.70	8.71–8.94	8.95–9.22	9.23–10.42	
n	207	231	171	198	
HFpEF, n (%)	11 (5.3)	27 (11.7)	23 (13.5)	45 (22.7)	*<0.001*
LVH, n (%)	47 (22.7)	87 (37.7)	61 (35.7)	104 (52.5)	*<0.001*
LV geometry					
Normal, n (%)	111 (53.6)	95 (41.1)	62 (36.3)	63 (31.8)	*<0.001*
Concentric remodeling, n (%)	49 (23.7)	49 (21.2)	48 (28.1)	31 (15.7)	
Eccentric hypertrophy, n (%)	24 (11.6)	41 (17.7)	26 (15.2)	41 (20.7)	
Concentric hypertrophy, n (%)	23 (11.1)	46 (19.9)	35 (20.5)	63 (31.8)	
LAD (mm)	37.79 ± 4.46	37.84 ± 4.89	38.18 ± 5.67	38.98 ± 4.98	*0.025*
AOD (mm)	32.73 ± 3.36	32.25 ± 3.41	32.39 ± 3.21	32.61 ± 3.14	0.42
LVIDD (mm)	47.45 ± 3.75	47.04 ± 4.83	46.95 ± 4.32	47.86 ± 4.27	0.177
LVIDS (mm)	29.67 ± 3.37	30.14 ± 3.64	30.13 ± 3.55	30.62 ± 3.77	0.072
IVSD (mm)	10.27 ± 1.45	10.85 ± 1.53	10.65 ± 1.60	11.13 ± 1.73	*<0.001*
PWTD (mm)	9.51 ± 1.25	9.92 ± 1.35	9.89 ± 1.20	10.09 ± 1.47	*0.001*
RWT	0.40 ± 0.06	0.43 ± 0.08	0.42 ± 0.06	0.42 ± 0.07	*0.002*
LVMI (g/m^2^)	94.11 ± 18.68	101.69 ± 21.65	101.00 ± 22.18	110.68 ± 27.90	*<0.001*
LVEF (%)	66.86 ± 5.33	65.73 ± 5.25	65.52 ± 5.81	66.12 ± 6.16	0.085
E/A	1.14 ± 0.20	1.10 ± 0.21	1.03 ± 0.21	1.03 ± 0.23	*<0.001*
DT (ms)	190.39 ± 61.03	203.78 ± 53.09	227.86 ± 47.37	246.88 ± 49.98	*<0.001*

Italic values represent* p* < 0.05

Compared to subjects in albumin-adjusted serum calcium quartile 1, those in quartile 4 had significant lower percentage of normal LV geometry (31.8 vs. 53.6 %); by contrast, percentage of the subjects with LV eccentric hypertrophy and concentric hypertrophy increased sharply from 11.6 to 20.7 %, 11.1 to 31.8 %, respectively (Table 3).

LAD, IVSD, PWTD, RWT, and LVMI of subjects in quartile 4 were significantly higher than those in quartile 1. As an indicator of systolic function, LVEF levels had no significant differences among groups categorized by albumin-adjusted serum calcium. E/A as well as DT, indicators of diastolic function, had significantly overall downward (1.14 ± 0.20 to 1.03 ± 0.23) and upward (190.39 ± 61.03 to 246.88 ± 49.98) tendencies, respectively, from quartile 1 to quartile 4 (Table 3).

### HFpEF

From albumin-adjusted serum calcium quartile 1 to quartile 4, percentage of the individuals with HFpEF increased sharply from 5.3 to 22.7 % (Table 3).

Besides the differences in albumin-adjusted serum calcium levels and clinical characteristics, echocardiographic parameters including LAD, LVIDD, LVIDS, IVSD, PWTD, RWT, LVMI, E/A and DT were also different between non-HFpEF and HFpEF group. In subjects with HFpEF, compared with eccentric hypertrophy (20.8 %), concentric hypertrophy (45.3 %) was the predominant abnormality in LV geometry (Table 1).

To determine the variables associated with HFpEF, backward stepwise multinomial logistic regression analysis was developed to include albumin-adjusted serum calcium, serum phosphate, age, gender, BMI, LVMI, SBP, DBP, TC, TG, HDL-C, LDL-C, smoking, lg Alb/Cr, lg HOMA-IR, uric acid and HbA1c on first step. HFpEF was significantly associated with albumin-adjusted serum calcium, old age, female gender, LVMI, lg HOMA-IR and uric acid (Table 4).

**Table 4:** Final model using backward stepwise multinomial logistic regression analysis to include albumin-adjusted serum calcium for HFpEF

	B	S.E.	Wald	df	p value	OR	95 % CI
Albumin-adjusted calcium	0.983	0.309	10.098	1	0.001	2.671	1.457–4.897
Age	0.064	0.012	27.076	1	<0.001	1.067	1.041–1.093
Gender	−0.672	0.234	8.269	1	0.004	0.511	0.323–0.807
LVMI	0.023	0.005	24.111	1	<0.001	1.023	1.014–1.033
Lg HOMA-IR	0.991	0.346	8.18	1	0.004	2.693	1.366–5.310
Uric acid	0.003	0.001	6.726	1	0.009	1.003	1.001–1.005

Variables entered on first step: Albumin-adjusted serum calcium, serum phosphate, age, gender, BMI, LVMI, SBP, DBP, TC, TG, HDL-C, LDL-C, smoking, lg Alb/Cr, HbA1c, lg HOMA-IR, uric acid

The binary logistic regression analysis (Table 5) showed the OR (95 % CI) for HFpEF according to changes in albumin-adjusted serum calcium concentration when calcium was a categorical variable (quartiles) or a continuous variable (per 1 mg/dL). In contrast to subjects in quartile 1 (8.42–8.70 mg/dL), there were significantly increased risk of HFpEF with subjects in quartile 4 [(9.23–10.42 mg/dL), OR (95 % CI) = 2.331 (1.088–4.994), p = 0.029], after adjusted for possible confounding factors including age, gender and obesity in model 1, further adjusted for smoking, hypertension, and dyslipidemia in model 2, and furthermore adjusted for LVMI, lg Alb/Cr, lg HOMA-IR and uric acid in model 3. When albumin-adjusted serum calcium level was analyzed as a continuous variable, the association between calcium and HFpEF maintained significantly in model 1, model 2 as well as model 3; and per 1 mg/dL increase, the OR (95 % CI) for HFpEF was [2.712 (1.471–5.002), p = 0.001] in the fully adjusted model.

**Table 5:** OR (95 % CI) of HFpEF according to albumin-adjusted serum calcium concentration

Model	Quartiles of albumin-adjusted serum calcium (mg/dL)				Continuous variable
	8.42–8.70	8.71–8.94	8.95–9.22	9.23–10.42	
n	207	231	171	198	807
Crude model	1.000 (reference)	2.358 (1.139–4.884), 0.021	2.769 (1.309–5.859), 0.008	5.241 (2.623–10.473), <0.001	4.945 (2.979–8.209), <0.001
Model 1	1.000 (reference)	2.349 (1.120–4.924), 0.024	2.514 (1.172–5.390), 0.018	4.824 (2.382–9.767), <0.001	5.295 (3.090–9.075), <0.001
Model 2	1.000 (reference)	2.433 (1.155–5.122), 0.019	2.520 (1.169–5.431), 0.018	4.813 (2.366–9.793), <0.001	5.021 (2.918–8.638), <0.001
Model 3	1.000 (reference)	1.722 (0.801–3.700), 0.164	1.657 (0.744–3.688), 0.216	2.331 (1.088–4.994), 0.029	2.712 (1.471–5.002), 0.001

Values are OR (95 % CI) and p value

Model 1: adjusted for age, gender and obesity

Model 2: further adjusted for smoking, hypertension, and dyslipidemia

Model 3: further adjusted for LVMI, lg Alb/Cr, lg HOMA-IR, uric acid

**Fig. 1:**
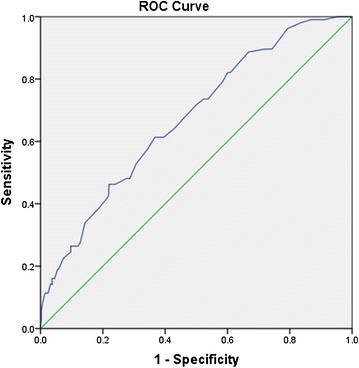
ROC curves of the ability of albumin-adjusted serum calcium to predict HFpEF

To evaluate the predictive performance of albumin-adjusted serum calcium for HFpEF, the AUC in ROC curve was calculated, which was 0.673 [95 % CI (0.620–0.726), p < 0.001] (Fig. 1).

## Discussion

Epidemiological studies have associated T2DM with HFpEF [5, 21, 22]. On the one hand, T2DM is a well-known trigger factor for HFpEF which exerts important effects on ventricular relaxation/stiffness [5] and coronary microvascular function [23]; on the other hand, diabetic cardiomyopathy is manifested by HFpEF other than HFrEF at an early stage [22]. Given that elevated serum calcium levels are associated with increased risks of T2DM [9, 10], an important question arises whether elevated serum calcium contributes to HFpEF prevalence in T2DM.

To the best of our knowledge, this is the first analysis of the relationship between changes in serum calcium levels and the risk of HFpEF that focused specifically on T2DM patients with normocalcemia and normophosphatemia. Our results showed a clear association between the elevated albumin-adjusted serum calcium levels and the increased risk of HFpEF. Such an association is independent of the effects of age, gender, obesity, smoking, hypertension, dyslipidemia, LVMI, HbA1c, Alb/Cr, HOMA-IR, and serum uric acid.

In our study, patients with HFpEF had significantly higher levels of albumin-adjusted serum calcium than those without HFpEF. On the other hand, patients in the highest serum calcium quartile had significantly greater percentage of HFpEF than those in the lowest quartile. Previous studies have demonstrated that higher serum calcium levels are associated with greater risks of incident HF [24], worse outcomes of HF [25], poorer clinical response to maximization of HF therapy [26]. However, the relationship between serum calcium and HFpEF is unknown. Our study indicates that elevated serum calcium though in normal range is related to HFpEF prevalence in T2DM.

Elevated serum phosphate concentrations have been associated with cardiovascular events including heart failure through its interactions with parathyroid hormone, vitamin D, and fibroblast growth factor 23 in some studies [27, 28]. In contrast, the third National Health and Nutrition Examination Survey showed that factors determining serum phosphate concentrations are largely unknown and previously observed associations of serum phosphate concentrations with cardiovascular events are unlikely to reflect differences in traditional cardiovascular risk factors [29]. Furthermore, serum phosphate have been more likey associated with HFrEF and eccentric hypertrophy rather than HFpEF and concentric hypertrophy [30, 31]. In line with these studies, our data do not support an association between serum phosphate and HFpEF.

LVH is associated with increased HF risk [22, 32]. Though eccentric hypertrophy can occur in HFpEF, ours and previous studies [3, 32] demonstrate that concentric hypertrophy is the common form of left ventricular structural abnormality observed in these patients. Furthermore, in patients with T2DM, serum calcium is associated with an increased risk of LVH [33]. In accord with these, patients in our study with concentric hypertrophy had a higher but not significant level of serum calcium (9.13 ± 0.40 vs. 9.05 ± 0.42 mg/dL, p = 0.083) and a greater percentage of HFpEF (28.7 vs. 16.7 %, p = 0.014) than those with eccentric hypertrophy; however, after adjusted for LVMI (the index for LVH), serum calcium remained significantly associated with HFpEF (Table 5). Hence, the association between serum calcium and HFpEF in patients with T2DM can somewhat, but not fully, be explained by LVH.

As a key pathophysiological mechanism of T2DM, insulin resistance is not only accompanied with an increase in intracellular calcium [34, 35], but also positively correlated with serum calcium level in ours and other studies [33, 36, 37]. Meanwhile, there is increasing awareness regarding the associations of insulin resistance with myocardial diastolic dysfunction, cardiomyopathy and heart failure [21, 38, 39]. In line with these studies, our results showed a significant correlation between the elevated lg HOMA-IR value and the increased risk of HFpEF [OR (95 % CI) = 2.693 (1.366–5.310), p = 0.004].

In the current study, the binary logistic regression analysis showed a significantly association between albumin-adjusted serum calcium and HFpEF. Moreover, consistent with previous studies, our final model using backward stepwise multinomial logistic regression analysis to include albumin-adjusted serum calcium for HFpEF showed that old age [3, 5], female gender [3, 5], uric acid [40], and HOMA-IR [21, 38, 39] were also related to an increased risk of HFpEF.

The potential mechanisms underlying association between serum calcium and HFpEF remains unclear; however, there are some possibilities. On the one hand, the elevation of serum calcium appears to function as a connecting link among various metabolic disorders. Ours and previous studies [33, 36, 37, 41–43] have demonstrated that serum calcium level was positively and linearly associated with glucolipid metabolic parameters including FPG, HOMA-IR, uric acid, TG, TC and LDL-C. Given that various metabolic abnormalities, such as diabetes [3, 5], obesity [3, 5], hyperuricemia [40], insulin resistance [21, 38, 39], and metabolic syndrome [21, 39, 44] have been reported in association with abnormal left ventricular diastolic function, which is the fundamental physiopathologic mechanism responsible for the development of HFpEF [45], the increased serum calcium level may correlate with HFpEF prevalence through metabolic abnormalities. On the other hand, serum calcium level has close relationship with some recognized pathological mechanisms of HFpEF [3] such as LVH [33] and vascular stiffness [11, 46]. Patients with HFpEF have a predominant abnormality in left ventricular distolic function [5], which is sensitive to disorders in calcium metabolism [47]. The increased diastolic tension is a result of elevated cytosolic diastolic calcium [8]. Abnormal calcium homeostasis is not only one of the mechanisms in HFpEF [8], but also a prominent feature in the transition from cardiac compensatory hypertrophy to heart failure [48].

Several limitations of this study should be noted. First, in our study no serum parathyroid hormone, vitamin D and fibroblast growth factor 23 levels are available for most of the patients which may help to delineate the underlying mechanisms for the association between serum calcium and HFpEF, so it is impossible to absolutely exclude potential confounding factors including primary hyperparathyroidism and secondary hyperparathyroidism due to vitamin D deficiency and/or renal insufficiency. To minimize these possibilities, we excluded individuals with serum calcium or phosphate levels outside the reference range. In addition, secondary hyperparathyroidism cannot account for the higher rate of HFpEF among patients with higher serum calcium in our study, because serum calcium levels are well-known lower or low-normal in individuals with secondary hyperparathyroidism. Second, the results are based on single serum calcium measurements; therefore, time course of changes in calcium is not available. Third, the majority of participants in our study were old Chinese, which may limit the generalizability of our results to other age-groups or ethnicities. Fourth, the sample size in this study is only moderate. Finally, the hospital-based cross-sectional study is vulnerable to sample selection bias and cannot establish a cause-effect relationship.

## Conclusions

Our results support the reported correlation between calcium and glucolipid metabolism, and extend previous findings of the association between serum calcium and cardiovascular disease, especially heart failure. The increased albumin-adjusted serum calcium level, within the physiological ranges, is independently associated with HFpEF prevalence in patients with T2DM.

## Abbreviations

HF: heart failure
HFpEF: heart failure with preserved ejection fraction
HFrEF: heart failure with reduced ejection fraction
LVEF: left ventricular ejection fraction
LVH: left ventricular hypertrophy
T2DM: type 2 diabetes mellitus
LAD: left atrial diameter
AOD: aortic root dimension
LVM: left ventricular mass
LVMI: left ventricular mass index
LVIDD: left ventricular internal diameter, diastolic
PWTD: posterior wall thickness, diastolic
IVSD: inter ventricular septum, diastolic
RWT: relative wall thickness
E/A: early to late mitral inflow velocity
DT: deceleration time
OR: odds ratio
CI: confidence intervals
HOMA-IR: homeostasis model assessment insulin resistance
Alb/Cr: urine albumin per gram urine creatinine
TC: total cholesterol
TG: triglycerides
LDL-C: low-density lipoprotein cholesterol
HDL-C: high-density lipoprotein cholesterol
FPG: fasting plasma glucose
BMI: body mass index
BSA: body surface area
SD: standard deviation
SBP: systolic blood pressure
DBP: diastolic blood pressure
